# Tribological Evaluation of Silica Nanoparticle Enhanced Bilayer Hydrogels as A Candidate for Cartilage Replacement

**DOI:** 10.3390/polym14173593

**Published:** 2022-08-31

**Authors:** Mohammad Mostakhdemin, Ashveen Nand, Maziar Ramezani

**Affiliations:** 1 Department of Mechanical Engineering, Auckland University of Technology, Auckland 1010, New Zealand; 2 Faculty of Engineering, University of Auckland, Auckland 1010, New Zealand

**Keywords:** acrylamide-alginate, acrylic acid hydrogels, bilayer nanocomposite hydrogels, silica nanoparticle, tribological properties, wear resistance

## Abstract

Polymeric hydrogels can be used as artificial replacement for lesioned cartilage. However, modulating the hydrogel formulation that mimics articular cartilage tissue with respect to mechanical and tribological properties has remained a challenge. This study encompasses the tribological evaluation of a silica nanoparticle (SNP) loaded bilayer nanocomposite hydrogel (NCH), synthesized using acrylamide, acrylic acid, and alginate via modulated free-radical polymerization. Multi-factor pin-on-plate sliding wear experiments were carried out with a steel ball counterface using a linear reciprocating tribometer. Tribological properties of NCHs with 0.6 wt% SNPs showed a significant improvement in the wear resistance of the lubricious layer and a low coefficient of friction (CoF). CoF of both non-reinforced hydrogel (NRH) and NCH at maximum contact pressure ranged from 0.006 to 0.008, which is in the order of the CoF of healthy articular cartilage. Interfacial surface energy was analysed according to Johnson, Kendall, and Robert’s theory, and NCHs showed superior mechanical properties and surface energy compared to NRHs. Lubrication regimes’ models were drawn based on the Stribeck chart parameters, and CoF results were highlighted in the elastoviscous transition regime.

## 1. Introduction

Articular cartilage (AC), coupled with viscous synovial fluid, dissipates imposed stresses at diarrheal joints [1]. The biphasic cartilage incorporated with chondrocytes and collagen fibers mitigates overstressing on the tissue [2,3]. The presence of horizontal collagen fibers in the superficial zone reduces the cartilage coefficient of friction (CoF) tremendously [4]. However, AC damage eventuates if the fibres’ crack growth rate, attributed to shear stress, exceeds cell repair [5]. Excessive pressure due to physiological activities or interstitial fluid deficiency because of aging yields cartilage lesions, resulting in a condition known as Osteoarthritis (OA). Cartilage is avascular; thus, deprived migration of chondrocytes slows down the process of self-recovery and causes severe pain on the damaged tissues [6,7]. It is worth mentioning that chondrocytes’ immobilization with loss of proteoglycans could affect AC thinning [8,9]. A practical approach to treat the damaged cartilage is total joint replacement, which is not recommended for younger patients due to its short service life (10–15 years) [10]. To address this issue, researchers have been exploring the development of artificial cartilage to postpone or eliminate the need for total joint replacement. Polymeric hydrogels have been studied vastly due to their resemblance to AC in mechanical and tribological properties [11,12,13]. Hydrogels are biologically favored materials because of their biocompatibility [14] with no toxic effects or stimuli of the immune system [15]. 

Forming tough hydrogels depends on monomer amalgamation resulting in various types of networks [16,17]. Monomer combination that achieves the desired mechanical properties include: acrylamide (AAm) that yields toughness in the network and resembles elastic properties of AC [18], acrylic acid (AAc) that bonds to crosslinkers and shortens polymer chains that result in a stiffer material as well as being hydrophilic and having high water sorption capacity [19], [2-(Methacryloyloxy)ethyl] trimethyl ammonium chloride (METAC) that reduces friction by retaining fluid in the hydrogel matrix [20], and alginate monomer that enhances the viscosity of the solution [21]. Mechanical strengthening of AAm hydrogels by nanoparticles (NPs) has been explored due to the NPs’ strong tendency to form bonds with the polymers’ hydroxyl groups [22]. Incorporating NPs resulted in swelling [23], reduced porosity [24], low toxicity, and excellent biocompatibility [24] in hydrogel systems. In contrast to other NPs, silica nanoparticles (SNPs) confirmed a sizeable effect on viscoelasticity and shear modulus, as it can restrain the polymer chains at the interphase of polymer and NPs [25]. SNPs provide extensive lock points within chains, which enhance the compressive strength of the material [26]. They also slow the relaxation behaviour and chain kinetics as they produce strong polymer-NPs bonds [27]. Polymer bonds loosen up if NPs are far from the chains [28]. Viscoelastic behaviour of the nanocomposite hydrogel (NCH) strengthened by SNPs has been investigated substantially, and it was shown that it closely resembles the mechanical behaviour of the AC [28,29,30]. Viscoelastic response of AC is time-dependent and is related to poroelasticity of the material [31,32]. 

Furthermore, earlier studies have demonstrated that SNPs not only enhance mechanical properties but also promote cell proliferation in hydrogels. On the other hand, PAAm-Alginate networks have been previously considered for biomedical applications due to their non-toxicity. SNP loaded PAAm-Alginate hydrogels, therefore, are attracting attention as biocompatible candidates for utilization in tissue engineering applications [27,33].

NCH loaded by SNPs showed adhesive wear as the main wear mechanisms, despite the fact that fatigue wear affected surface pitting as the applied normal load increases [33]. Adding 1 wt%–4 wt% SNPs in the PAAm-Alginate polymer network resulted in a low CoF of around 0.0035–0.0055 [33]. This is because of a robust entanglement of polymer-NPs interface inside the hydrogel matrix [33], which resemble AC (0.001) [34]. Essentially, contact stress and pore pressurization inside linked channels are the main elements that control the hydration levels during sliding wear tests [35]. The contact stress carried by AC is in the range of 0.1–2.0 MPa in the knee and hip joints [34,36]. By intensifying contact pressure, the CoF reduces in AC [37,38]. However, research showed that testing factors and rehydration would alter the tendency of the material to show lower CoF at higher contact pressures [39]. Jayanth et al. [38] showed that CoF decreases by increasing the contact stress from 0.2 to 0.5 MPa. CoF value depends on many factors in hydrogels, such as hydrophilic-moulded synthesizing process [40], types of ionic crosslinking [20], lubricant types [41], and crosslinking density [13]. Crosslinking density is equivalently associated with the mesh size and showed an extraordinary link with the transition between low and high frictions [42]. However, lubrication mechanisms of hydrogels have not been investigated in detail. The influence of contact stresses and sliding speeds on the lubrication regimes does not follow the standard engineering Stribeck curve [43]. Lubrication has been presented in three regimes, i.e., mesh-confined regime, elastoviscous transition regime, and fluid film lubrication regime. SNPs impact on mesh shapes, and thus, are convincingly associated with the lubrication regimes [33]. Due to the hydrogels’ conformational surface and its poroelastic response, assessment of hydrogels in the viscoelastic transition regime remained mostly intact [44].

AC lubrication depends on several factors, including (1) rehydration (2), contact stress, (3) cartilage friction and wears mechanisms [45,46,47]. Therefore, key parameters necessary to assess artificial cartilage are lubricant, contact pressure, sliding velocity, and hydrogel network structure. Promoting the mechanical strength can negate the tribological performance and vice versa [48]. Thus, developing a hydrogel with sufficient load-bearing capacity and superior tribological properties is the main focus of the current research. 

Studying the wear resistance and CoF of hydrogels has been discussed extensively; however, utilizing SNPs to enhance tribological properties in the lubricious layer has not been investigated. Hence, in this paper, the assessment of tribological properties and dominant wear mechanisms and identification of the lubrication regimes are discussed for our developed bilayer silica-nanocomposite hydrogels (NCHs). The significance of our research is the development of a hydrogel with high mechanical and tribological performances and maintaining both properties at the desired level for cartilage replacement applications.

## 2. Materials and Methods

### 2.1. Materials Preparation

**Figure 1:**
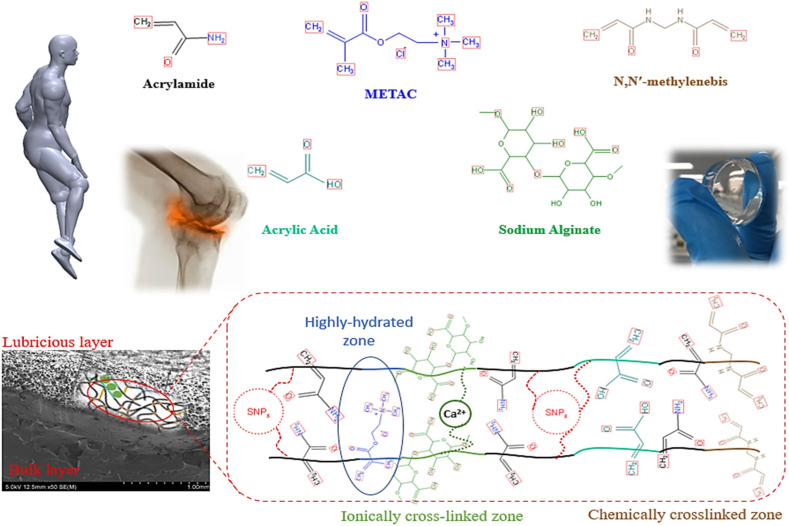
Utilized materials and IPNs structure of the NRH and NCHs.

PAAm-PAAc-Alg-METAC hydrogel was synthesized via modulated free radical polymerization as described previously [49]. For chemical crosslinking, the total monomer: crosslinker mole ratio was maintained at 490:1. As stated in our previous report [49], the monomer/ SNPs/ crosslinker mixtures were allowed to cure for 24 h and 35 °C constant temperature to facilitate the modulated free radical polymerization. Cured hydrogels were then immersed in 16.0 *w*/*v*% CaCl_2_ solution for 24 h to form the secondary networks through ionic crosslinking. All materials were acquired from the Sigma Aldrich company (St. Louis, MO, USA) and utilized without any additional purification. Probe sonication was used to disperse the SNPs in DI water for 25 min and then added to the prepared monomer mixture, prior to curing, under mechanical stirring. Four different SNP concentrations (0.05 wt%, 0.2 wt%, 0.4 wt%, and 0.6 wt%) were loaded in the hydrogel matrix, and the influence of the SNPs concentrations on the mechanical properties of the NCHs was studied [49]. A sample with the best mechanical properties, with optimized crosslinking density, was chosen for the tribological performance assessment. All mentioned concentrations of SNPs in NCHs were evaluated and compared with the NRHs, which is the hydrogel without the SNPs fillers. Figure 1 represents the materials utilized in the proposed system.

### 2.2. Tribology Tests

Sliding wear experiments were carried out by a linear reciprocating tribometer (Rtec-instruments, San Jose, CA, USA). Multi-factor experiments were designed and conducted in this paper as presented in Table 1. Set 1 aimed to focus on load factor with a constant sliding speed, while set 2 focused on the influence of the sliding speeds under a constant normal load. Three loads of 0.5, 0.7, and 0.9 N were considered to impose a range of contact pressures experienced by AC in daily activities [34,36,50]. Pressure-sensitive films (prescale LLLW and LLW ranges) obtained from Fujifilm were placed between contacting counterface and hydrogels to measure the contact pressure on hydrogel implants. The equivalent contact pressure is a function of the samples’ modulus of elasticity. The applied normal loads of 0.5–0.9 N resulted in contact stresses of 0.210–4.50 MPa, and 0.305–4.25 MPa for NRHs and NCHs strengthened with SNPs, respectively. Three sliding frequencies (3.125, 5, and 6.875 Hz) with 8 mm sliding stroke were set to output 50 mm/s, 80 mm/s and 110 mm/s sliding speeds. These speeds have been reported as walking, jogging, and running sliding speeds in AC [34]. A 4 mm diameter ball made of stainless steel was used as the sliding mate for all sliding wear tests. Two 10 N load-cells attached to the tribometer’s probe recorded forces in vertical and horizontal directions to calculate CoF. All specimens were placed in an incubator room with a controlled temperature of 35 °C to dry them thoroughly and then inserted into a custom-designed jig immersed in DI water three days prior to each test. The hydrated samples were used for the tribology tests. All sliding wear tests were carried out in pin-on-plate mode with linear reciprocating motion. Based on the mentioned frequencies, a series of times were programmed to run all tests for 1000 m sliding distance. Table 1 presents all experimental factors that have been set in sliding wear tests.

**Figure 2:**
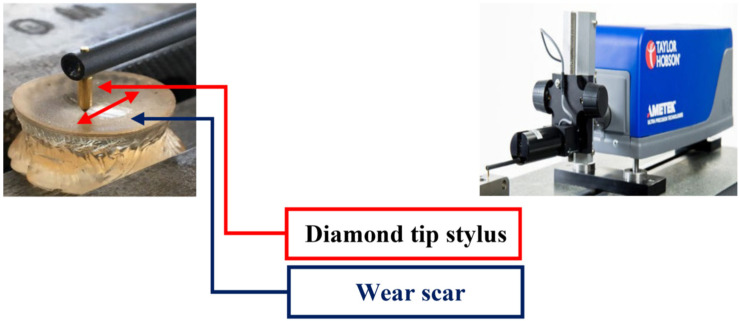
Stylus profilometer to measure the wear scars.

Upon completion of each sliding wear test, the wear profile was measured by a stylus profilometer (Taylor Hobson, AMETEK, PA, USA) equipped with a diamond tip to measure the wear scars’ depth and width. Three different cross-sections of each wear track were scanned by the profilometer to plot the wear profiles. The plotted graphs were then processed by ImageJ software (National Institute of Health, Bethesda, MD, USA) to calculate the wear surface area. After this stage, the average of three measured surface areas was multiplied by 8 mm (length of sliding stroke) to calculate wear volume. Figure 2 illustrates the measurement of wear scars.

### 2.3. Scanning Electron Microscopy

A field emission scanning electron microscope (Hitachi, SU-70 FE-SEM, Tokyo, Japan) was employed to observe the worn off surface to figure out the wear mechanisms after sliding tests. After obtaining the wear profiles by the stylus profilometer, specimens were kept in the incubator room at a constant temperature of 35 °C to dry the samples thoroughly for 240 h. Dried samples were cut to shrink the size of samples by grinding with mild-rough sandpaper. It is noteworthy that the wear track remained intact and untouched, and only the sides of the samples were cut. Then, all samples, contained in a plastic tube, were immersed in liquid nitrogen for 10 minutes to gradually lyophilize the structure. After this stage, hydrogel samples were kept for 72 h in a freeze-dryer at 10 µBar to dry the entire network. Subsequently, hydrogel specimens were coated with platinum powder with a sputter coating machine (Hitachi E-1045, Tokyo, Japan) set at 25 mA for 100 seconds. Lastly, hydrogel specimens were attached to a metallic jig and placed in SEM with 5.0 kV power to acquire images of worn off surfaces and bed with different magnifications. 

### 2.4. Statistical Analysis

All hydrogel samples were tested at least three times, and results were obtained as mean ± standard deviations. A two-way ANOVA was employed for statistical analysis of the results. Post hoc Tukey tests were performed to govern the statistical significance of the results obtained for NRH and NCH test specimens with 95% confidence. Some parameters such as the applied loads, sliding speeds, and material types (NRHs and NCHs) were set as inputs in the statistical analysis to study the effects of each on the wear rate and CoF. 

## 3. Results and Discussions

### 3.1. Coefficient of Friction

**Figure 3:**
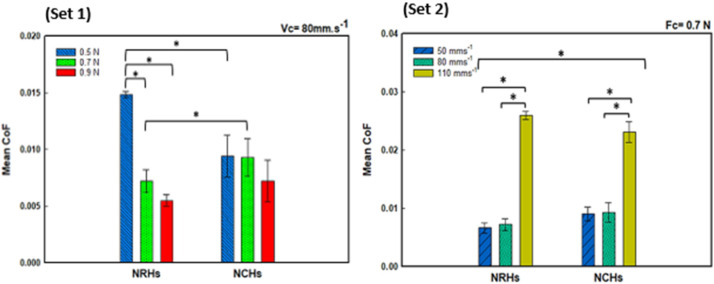
Mean values of the coefficient of friction for NRHs and NCHs samples in lubricated condition versus (set 1) applied load (Vc = 80 mm/s), and (set 2) sliding speed (Fc = 0.7 N), (n = 3 ± SD). * ANOVA and post-hoc Tukey tests (*p* < 0.05) were conducted for statistical significance analyses.

Sliding wear experiments were carried out with NRHs and NCHs samples with 0.6 wt% NPs concentration exhibiting superior mechanical properties. Figure 3 demonstrates that CoF mean values in lubricated conditions decreased with increasing applied load. In NCHs, the imposed load of 0.5 N resulted in contact pressure of 0.305 MPa, close to the lower range of contact pressure experienced by AC (≈ 0.1MPa) [34,36,50]. Under this applied load, the mean CoF value was around 0.009, as presented in Figure 3 (set 1). The attained CoF values are comparable to that of cartilage at 0.001 [34]. Under 0.7 N load, a 50% drop in CoF was observed in NRHs, which is because of the conformed region under the sliding probe due to its lower stiffness than NCHs. Therefore, the accumulated fluid reduces the shear forces of contacting asperities and lowers the CoF. In NCHs, due to a firmer lubricious network and smaller porosity sizes, insignificant fluid diffuses out. Then it boosts up asperity adhesion and maintains CoF at the same level. Geong and Osada [51] reported that CoF magnitude is highly contingent on the repulsion-adsorption friction. A high water level is retained at a high-load regime due to its electrostatic repulsion, and therefore, it yields to lower frictional forces between contacting asperities.

The influence of the sliding speed (at a constant normal load of 0.7N) on CoF of both NRHs and NCHs samples is shown in Figure 3 (set 2). Results for the sliding speeds of 80 mm/s are not shown as they are already presented in set 1. NCHs exhibited lower CoF in 50 mm/s and 110 mm/s. It has been reported that SNPs in the lubricious layer affect polarity and surface tension, subsiding CoF [52]. Short strands at low sliding speeds result in lower CoF values [53], since SNPs enhance crosslinking density. SNPs also promote abrasion resistance because they shorten the length of dangling chains and reduce residual stress and shrinkage [54].

**Figure 4:**
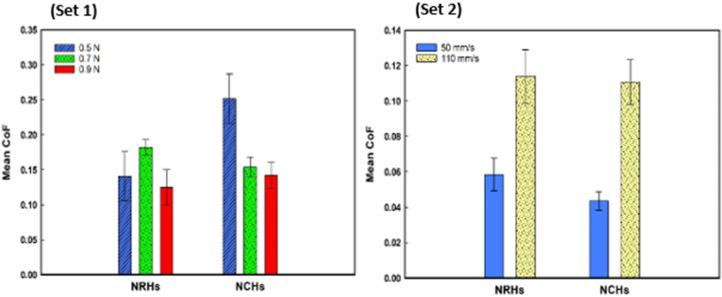
Mean values of the coefficient of friction for NRHs and NCHs samples in dry condition versus (set 1) applied load (Vc = 80 mm/s), and (set 2) sliding speed (Fc = 0.7 N) in dry condition, (n= 3 ± SD).

Figure 4 illustrates the mean values of the CoF for NRHs and NCHs samples tested in dry contact. The lubricious layer of the bilayer hydrogel samples incorporated with METAC monomer would retain water and significantly impact CoF. Hence, tribology tests were conducted in dry conditions to test this hypothesis. NRHs showed different patterns compared to NCHs. By increasing load to 0.7 N, matrix stiffness in NRHs conformed and due to loose mesh sizes, network destructions resulted in weaving dangling chains; thus CoF values increased. At 0.9 N load, CoF decreased in NRHs due to the entire compression of the lubricious layer by the contacting mate, which promptly deteriorated it. Therefore, the sliding probe mainly slides over the bulk region with a firmer matrix and lower CoF. In NCHs, a continuous decrease by increasing load is because of a firmer stiffness through the thickness in the lubricious layer and its sufficient resistance against a higher contact pressure. In set 2, sliding speed variations highlight the adhesion between counterparts. From 50 mm/s to 110 mm/s, a lower CoF threshold in NCHs shows SNPs effect in reducing the adhesive strength of dangling chains.

In dry conditions, the hydrogel matrix stiffness is a function of the SNPs, mesh size, the conformational volume of the wear track, and fluid loss due to generated heat. Furthermore, elastic force and its distribution on surface energy are correlated to the adhesion variations and CoF. Robert and Kendall used rubber and glass contact in their experiments and reported that at a low-load regime, strong adhesion was found when the surfaces were dry. Therefore, the Johnson–Kendall–Roberts (JKR) model was used to estimate surface energy, adhesion, and contact area to address friction values, especially in the absence of lubricant in our proposed bilayer system [55]. Due to different crosslinking densities, dangling polymer chains, and porosity architecture presented in NRHs and NCHs, we assumed stored elastic energy and lost surface energy between asperities that came into contact. Therefore, consideration of the contact equilibrium from elastic matings requires considering total energy *U_T_* as a function of the radius of contact. In the system, total energy *U_T_* is the total of the elastic energy *U_E_* stored in the system, the mechanical energy *U_M_* from the applied load, and the surface energy *U_S_*.

(1) $$U_{T}=U_{E}+U_{M}+U_{S}\;$$

The surface energy *U_s_* is defined as below:

(2) $$U_{S}=-\pi a_{1}{}^{2}\gamma \;$$

where *a* is the radius of the circle of contact region, and *γ* is the energy per unit contact area that is obtained by:

(3) $$\gamma =\frac{P_{0}\left(R_{1}+R_{2}\right)}{\pi R_{1}R_{2}}\;$$

where *P*_0_ is the normal load imposed on the hydrogel, *R*_1_ is the radius of the contacting indenter, and *R*_2_ is the radius of the deformed hydrogel under the contacting load.

(4) $$a_{1}{}^{3}=RP_{1}/K\;$$

Elastic contacts of indenter and hydrogel are calculated as below:

(5) $$k_{1}=\frac{1-v_{1}^{2}}{\pi E_{1}}\;,\;k_{2}=\frac{1-v_{2}^{2}}{\pi E_{2}}$$

where *v* is the Poisson’s ratio and *E* the Young’s modulus of each material. All of the mentioned terms are presented in Table 2 at the end of this section. From Equation (4), *R* and *K* can be obtained by $R=R_{1}R_{2}/(R_{1}+R_{2})$ and $K=4/3\pi \left(k_{1}+k_{2}\right)$. Thus, in Equation (2), the surface energy is obtained as below:

(6) $$U_{S}=-\pi \gamma {\left(\frac{RP_{1}}{K}\right)}^{\frac{2}{3}}\;$$

**Figure 5:**
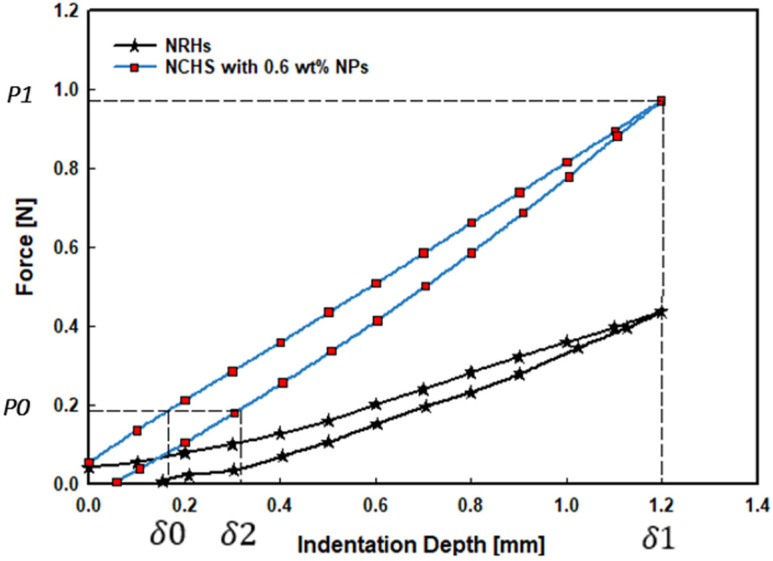
Load-displacement graphs for NRHs and 0.6 wt% loaded NCHs.

The elastic energy *U_E_* is the difference between imposing energy to the system *U_1_* and releasing energy from the system *U_2._* The attained graphs for both NRHs and NCHs are shown in Figure 5.

Therefore, the stored elastic energy is:

(7) $$U_{E}=U_{1}-U_{2}\;$$

Neglecting surface forces, load *P*_1_ was imposed to form the contact radius of *a*_1_, which requires energy *U*_1_.

(8) $$U_{1}=\int_{0}^{P_{1}}\frac{2}{3}\frac{P^{\frac{2}{3}}}{K^{\frac{2}{3}}R^{\frac{1}{3}}}dP=\frac{2}{5}\frac{P_{1}^{\frac{5}{3}}}{K^{\frac{2}{3}}R^{\frac{1}{3}}}\;$$

Keeping the contact radius at *a*_1_, the load then decreased to *P*_0_ to give the system’s final state, releasing energy *U*_2_.

(9) $$U_{2}=\int_{P_{0}}^{P_{1}}\frac{2}{3}\frac{P}{Ka_{1}}\;dP=\frac{1}{{3K}^{\frac{2}{3}}R^{\frac{1}{3}}}\left[\frac{P_{1}^{2}{-P}_{0}^{2}}{P_{1}^{\frac{1}{3}}}\right]\;$$

Therefore, substituting Equations (8) and (9) into Equation (7), the elastic energy equation is attained as follows:

(10) $$U_{E}=\frac{1}{K^{\frac{2}{3}}R^{\frac{1}{3}}}\left[\frac{1}{15}P_{1}^{\frac{5}{3}}+\frac{1}{3}P_{0}^{2}P_{1}^{-\frac{1}{3}}\right]\;$$

The mechanical potential energy *U_M_* of the applied load *P_0_* is:

(11) $$U_{M}=-P_{0}\delta_{2}\;$$

where *δ* is the elastic displacement and can be obtained by:

(12) $$\delta =\frac{2}{3}P/Ka_{1}\;$$

Thus, the final mechanical energy *U_M_* is:

(13) $$U_{M}{=–P}_{0}\left[\delta_{1}–\frac{2}{3}\frac{\left(P_{1}{-P}_{0}\right)}{{Ka}_{1}}\right]=\frac{-P_{0}}{K^{\frac{2}{3}}R^{\frac{1}{3}}}\;\left[\frac{1}{3}P_{1}^{\frac{2}{3}}+\frac{2}{3}P_{0}P_{1}^{-\frac{1}{3}}\right]\;$$

Substituting Equations (6), (10) and (13) into Equation (1) results in total energy *U_T_*

(14) $$U_{T}=\frac{1}{K^{\frac{2}{3}}R^{\frac{1}{3}}}\left[\frac{1}{15}P_{1}^{\frac{5}{3}}+\frac{1}{3}P_{0}^{2}P_{1}^{-\frac{1}{3}}\right]-\frac{1}{K^{\frac{2}{3}}R^{\frac{1}{3}}}\;\left[\frac{P_{0\;}P_{1}^{\frac{2}{3}}}{3}+\frac{2}{3}P_{0}P_{1}^{-\frac{1}{3}}\right]-\gamma \pi \frac{R^{\frac{2}{3}}P_{1}^{\frac{2}{3}}}{K^{\frac{2}{3}}}\;$$

Table 2 presents variables used to calculate interfacial surface energy for samples. The total energy in NCHs increased by up to four-fold compared to NRHs. Results showed that even by a higher amount of surface, mechanical, and stored elastic energies in NCHs, which indicates enhanced adhesion strength, CoF values in dry conditions were reduced compared to NRHs. It presents that NCHs strengthened with SNPs significantly improved mechanical and tribological performances compared to NRHs.

**Figure 6:**
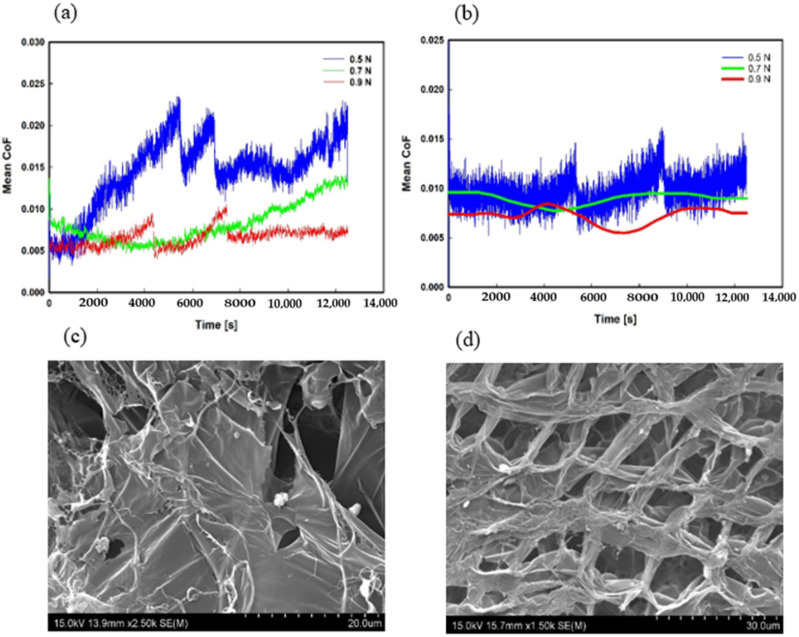
Variations of CoF versus time for 1000 m sliding wear tests at 0.5 N, 0.7 N, and 0.9 N; in (**a**) NRHs and (**b**) NCHs. SEM images of the superficial layer in (**c**) NRHs and (**d**) NCHs.

The variations of CoF versus time for both NRHs and NCHs are shown in Figure 6. CoF in NRHs, under 0.5 N load, fluctuated during the sliding wear tests. It indicates the instability of the network resistance against shear forces. A continuous increase up to 5500 s and a sudden fall demonstrate shear stresses overcome the network strength. The SEM image of the lubricious layer for NRHs shows irregular and weak bonding that yields unstable resistance showed in Figure 6a. In contrast, Figure 6b illustrates reasonable stability in network resistance after being topped up with 0.6 wt% SNPs. The SEM image of NCHs in Figure 6d presents a very regular and firm mesh network, which averts the lubricious layer destruction in the early stages. It is worth mentioning that lines for NCHs in Figure 6b for both 0.7 N and 0.9 N loads were smoothed to a single line for the better graph presentation since all results fall in the minimum CoF threshold. SEM images, along with CoF trends, ascertain the prominence of the size of the linkages and meshes. It is clear that the optimum size of crosslinked chains detrimentally minimizes the network destruction rate. It is because of the improvement in chains transverse micromotions. A sudden fall in CoF could be due to the unlinked spaces in meshes that are present in NRHs. It has been reported that transverse micromotions of linkages steadily increase the CoF until the breaking point due to their material loss [56]. 

### 3.2. Wear Volume

**Figure 7:**
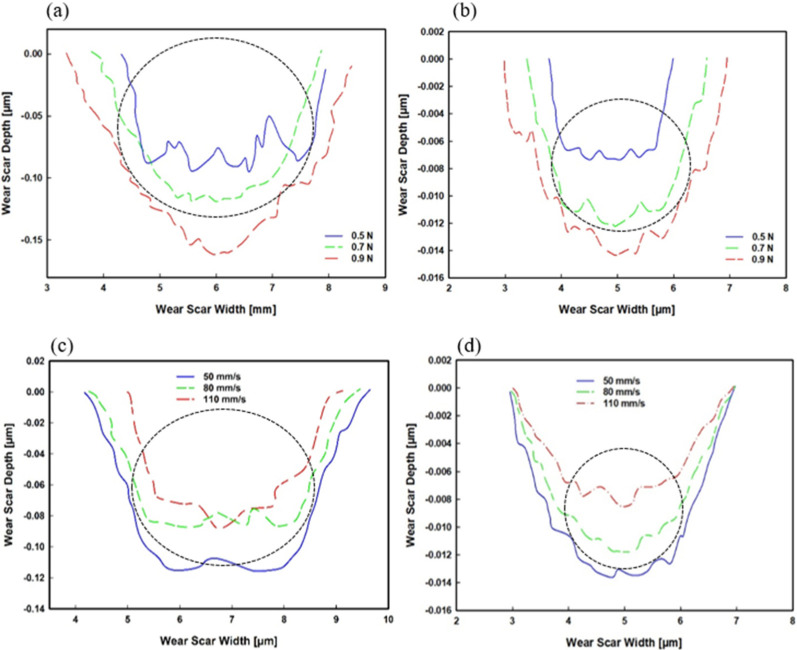
Wear scar depth and width for different applied normal loads under a constant sliding speed of 80 mm/s for (**a**) NRHs, (**b**) NCHs samples; and at different sliding speeds under a constant load of 0.7 N for (**c**) NRHs, (**d**) NCHs samples.

The stylus profilometer was utilised to scan the wear tracks and plot their depths and widths after each sliding wear test. The variations of wear profiles under different loads and sliding speeds are presented in Figure 7. At 0.5 N applied load in NRHs, a severely chattered profile was obtained compared to NCHs, indicating unlinked porosity through the lubricious layer’s thickness in NRHs. However, a flatter and narrower profile in NCHs shows well-distributed crosslinking, illustrated in Figure 7b.

Overall, the wear profiles in NRHs tested under 0.7 N and 0.9 N applied loads are more sweeping than NCHs. This phenomenon is because of the network elastic energy absorbed by the pressure from the contacting ball. The greater elastic energy in NCHs resulted in a lower transverse deformation imposed by contacting mate. By increasing speed, the worn area decreased because of the adhesion tendency between contacting mates. As the sliding speed increases, less adhesion strikes between contacting asperities and, therefore, less material loss, as reported by Kim et al. [57]. This phenomenon becomes prominent with the abundance of long-length dangling chains in the superficial layer in NRHs. 

In NCHs, the worn profile gradually decreased with increasing sliding speed; however, in NRHs, wear depth remained intact from 80 mm/s to 110 mm/s. This shows that wear in NRHs is independent at higher sliding speeds, whereby adhesion strength would not decrease. In both samples, wear depth did not exceed 15 µm, which is much lower than the thickness of the lubricious layer (235 µm in NRHs and 485 µm in NCHs).

**Figure 8:**
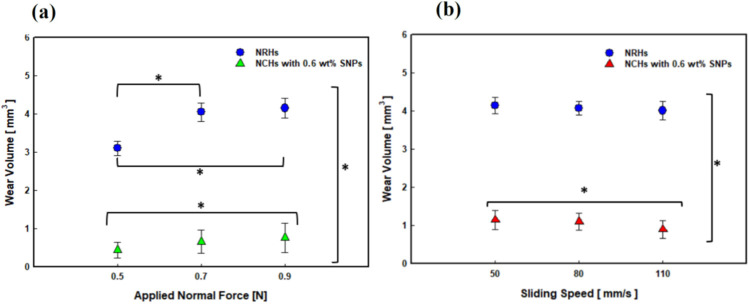
Wear volume of NRHs and NCHs samples at different (**a**) loads and (**b**) sliding speeds (n = 3 ± SD). All tests were performed in lubricated contact. * Statistical significance analyses were conducted by ANOVA and post hoc Tukey test (*p* < 0.05).

The wear volumes of samples are presented in Figure 8. Increasing load resulted in increased volume of wear in both NRHs and NCHs, as shown in Figure 8a. However, introducing 0.6 wt % SNPs strengthened up the wear resistance of the NCHs and its wear volume is less than one-third of the wear volume in NRHs. By increasing sliding speed, wear volume overall decreased in both NRHs and NCHs. Significantly improved wear resistance in NCHs was due to lessening of adhesion tendency and higher elastic energy as described in the previous section. Our formulation shows significant wear loss reduction by utilizing additional monomers (AAc, METAC) and SNPs. AAc incorporation with SNPs reported enhanced tensile properties and 900% elongation at the break point [58], and it also showed SNPs were masked by polymer chains and shaped core-shell structures [59], which produce a firmer structure for sliding contacts.

### 3.3. Wear Mechanisms

**Figure 9:**
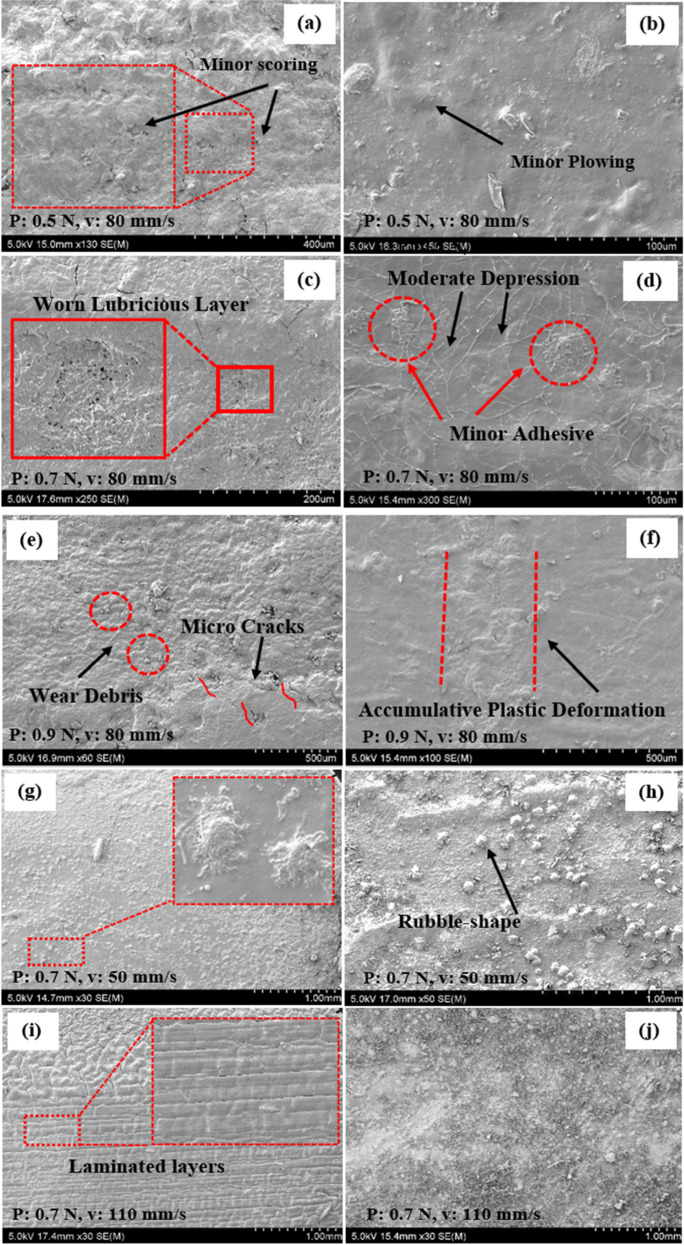
SEM micrographs of the wear regions for samples tested under different loads and sliding speeds in (**a**,**c**,**e**,**g**,**i**) NRHs samples and in (**b**,**d**,**f**,**h**,**j**) NCHs samples. All sliding wear tests were carried out in lubricated contact condition.

In lubricated contact, at the applied normal load of 0.5 N in NRHs samples, minor scoring was observed in Figure 9a, and this is due to network flaws or loops associated with self-bonding or crosslinking deficiency. Figure 9b, presents NCHs, showing an integrated and connective surface. Minor ploughing improved polymer plastic deformation with the same experiment set compared to NRHs. By increasing the normal load to 0.7 N, significant distinctions are elucidated in the wear mechanisms of NRHs and NCHs. NRHs showed the worn lubricious layer due to the moderate adhesive wear mechanism in Figure 9c. It indicates the brittle fracture of dangling chains and dysconnectivity of the network, as also discussed in the recent research study [60]. In contrast, a minor adhesive patch and moderate depression in NCHs occurred due to the enhanced plastic deformation in strands in Figure 9d. It is evident that by increasing the applied load from 0.5 N to 0.7 N, in NRHs samples, the wear mechanism transformed from abrasive to adhesive phase. In contrast, in NCHs, the accumulative plastic rate only increased without evidence of disintegrated tissues. At 0.9 N load, as illustrated in Figure 9e, microcracks and wear debris were spotted in NRHs specimens, and the superficial surface of the samples depreciated. By increasing the load from 0.7 N to 0.9 N, the presence of wear debris and cracks in Figure 9e indicates that the primary wear mechanism shifted from adhesive wear to fatigue wear. This observation implies that the wear mechanism of the PAAm-PAAc hydrogel is load-dependent. However, in NCHs, moderate accumulative plastic deformation was observed, which shows wear mechanism remains mostly intact compared to lower load magnitudes in Figure 9f. Utilizing SNPs leads to the load-independent wear mechanism of the NCHs network.

By maintaining constant load and slowing down the sliding speed to 50 mm/s, convex-shape debris on the surface of NRHs appeared, and rubble-shape particles on the surface of NCHs illustrated in Figure 9f,g, respectively. In NCHs, shorter strands were debonded, rolled under the sliding ball, and formed rubble-shape particles. Comparing NCHs with the same applied normal load and higher sliding speed (*p* = 0.7, v = 80 mm/s) in Figure 9d, the adhesive patches contain tiny spheres on the surface. When sliding speed is 80 mm/s, adhesion between asperities stuck them up in sparser zones. On the other hand, when the speed decreased to 50 mm/s, contacting asperities had sufficient time to detach spheres, roll them over, and distribute them randomly due to a longer test period. Increasing sliding speed to 110 mm/s resulted in a flattened superficial layer in NRHs with laminated layers along the sliding direction. Conversely, in NCHs, the wear track is barely recognized since the prompt movement of the sliding ball and lessened adhesion minimized asperities engagements and the wear loss.

### 3.4. Lubrication Mechanisms

**Figure 10:**
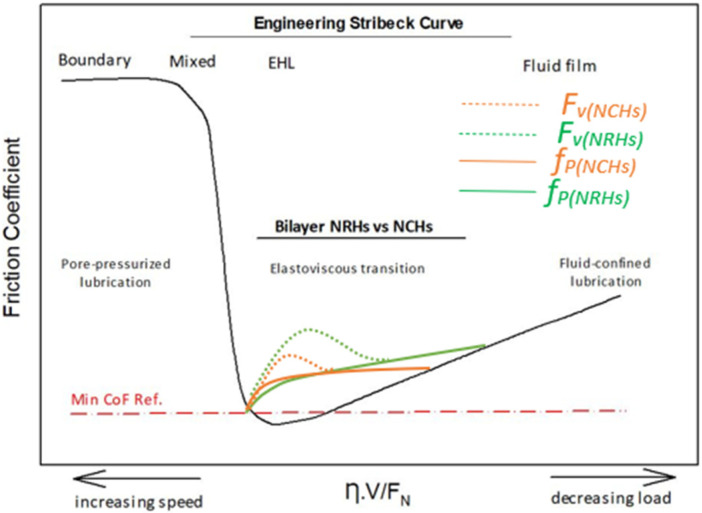
Schematic illustration of developed lubrication regimes in hydrogels in the frame of the engineering Stribeck curve.

Hydrogels are viscoelastic and constant pressure was maintained during the sliding wear tests; therefore, boundary lubrication and fluid film lubrication regimes are not relevant. While it is known that hydrogel materials do not adhere strictly to the Stribeck engineering curve [44], three main regimes have been developed for hydrogels: pore-pressurized lubrication regime, elastoviscous transition regime, and fluid-confined lubrication regime, as shown in Figure 10. According to our experimental setup, the elastoviscous regime has been focused and models were developed with the aid of load and sliding speed factors. Boosting up speed reduces CoF, as the adhesion increases at lower applied loads and higher sliding speeds. Under this lubrication regime, fluid was maintained and confined in the network, resulting in the lubricious layer to remain intact, as less adhesion takes place between asperities because of the high sliding motion between the hydrogel sample and the counter material. Thus, at his phase, water retention rate and mesh size significantly affect the tribological properties like CoF and wear volume. At maximum contact pressure (4.25 MPa), a large amount of water would pump out due to high conformal contact, which results in less adhesion between contacting asperities in NCHs observed in Figure 9f. This phenomenon results in lower shear stress and CoF, especially for NCHs with shorter strands.

In contrast, in NRHs, long dangling chains could be woven during the reciprocating movement, resulting in a sudden-detached region as shown in Figure 9e. Because of the described phenomenon, reciprocating movement initiates dynamic shear, and also due to the fluid viscosity alteration by mixing with polymer debris, elastoviscous transition (EVT) occurs [44]. Furthermore, by decreasing load, a large amount of water traps in the interconnected channel and does not diffuse out, which results in a less conformal-hydrated region with an increased CoF. This regime in hydrogels is called fluid-confined lubrication (FCL). That is why, by moving from EVT to the FCL regime, CoF increases. SNPs detrimentally play an important role in the transition of EVT to the FCL regime, since a gradual increase in CoF was drawn in the NCHs samples, compared to the NRHs samples. By considering the speed factor (F_v_), less CoF magnitude variation was mapped for NCHs. F_v_ and *f_p_* stand for speed and load factors, respectively. It is noteworthy that the lubrication mechanism of the AC is significantly related to its permeability and the shear-thinning viscosity of the synovial fluid. The viscosity has a crucial role in maintaining a thin film of the lubricant that detaches the cartilage contacts and reduces the applied load [61].

## 4. Conclusions

In summary, this study concentrated on developing advanced NCHs with a lubricious layer by which tribological properties improved compared to recent developed hydrogels for cartilage replacement application. The following conclusions can be drawn as significant from our study:

- Regarding tribological properties, wear resistance in the NCHs samples improved significantly, and lower CoF was observed compared to the NRHs samples.
- With respect to the lubricious layer topography and its worn surfaces, in the control sample, by increasing the load, wear mechanisms were transformed from abrasive and adhesive to fatigue phase. However, in NCHs, wear mechanisms remained intact and mostly accumulative plastic deformation was observed.
- By adding SNPs, a four-fold increase in calculated total surface energy of NCHs were achieved compared to NRHs, which remarkably affected the improvement of tribological properties.

## Figures and Tables

**Table 1:** Multi-factor sliding wear tests.

Experiments Sets	Test Parameters		Contact Conditions
Set 1 (NRHs and NCHs)	0.5 N	Constant sliding speed [80 mm/s]	Dry and Lubricated
	0.7 N		
	0.9 N		
Set 2 (NRHs and NCHs)	50 mm/s	Constant load [0.7 N]	
	80 mm/s		
	110 mm/s		

**Table 2:** Variables used for total energy calculations of hydrogel samples.

Variables	*NRHs*	*NCHs*
*Elastic modulus (E)*	150 kPa	240 kPa
*Poisson’s ratio (v)*	0.5	0.5
*R*	0.7 mm	0.5 mm
*P*_0_	0.1 N	0.2 N
*P*_1_	0.4 N	1.0 N
*δ*_1_	1.2 mm	1.2 mm
*δ*_2_	0.4 mm	0.35 mm
*δ*_0_	0.2 mm	0.18 mm
*U_S_*	34.22	170.88
*U_E_*	11.28	62.34
*U_M_*	0.035	0.164
*U_T_*	**22.97**	**108.71**

## Data Availability

Data presented in this study are available upon request.
